# The CTLA4 -819 C/T and +49 A/G dimorphisms are associated with Type 1 diabetes in Egyptian children

**DOI:** 10.4103/0971-6866.45001

**Published:** 2008

**Authors:** Hatem Mohamed Saleh, Nestor Rohowsky, Michael Leski

**Affiliations:** Genotyping Lab for Genetics of Type One Diabetes- Autoimmune Diseases Section, R and D Department, The Egyptian Organization for Biotechnology (EGYTEC) - The Egyptian Organization for Biological Products and Vaccines (EGYVAC-VACSERA), 1 El Batal Ahmed Abdel Aziz Street, Agouza, Cairo 1312, Egypt; 1 Advanced Life Sciences, 1440 Davey Road, Woodridge, IL, U.S.A. 60517

**Keywords:** CTLA-4, Egyptian population, genotyping, mutation, single nucleotide polymorphisms, T1D

## Abstract

**BACKGROUND:**

Type 1 diabetes (T1D) is an organ-specific autoimmune disease characterized by T cell-mediated destruction of pancreatic islets. T cell proliferation is negatively regulated by cytotoxic lymphocyte antigen-4 (CTLA-4). CTLA-4 polymorphisms are associated with T1D in some but not all populations.

**AIMS:**

The study was conducted to investigate the association of the C-819T and A+49G single nucleotide polymorphisms (SNP) of CTLA-4 gene in T1D patients in the Egyptian population.

**METHODS:**

The association of the C-819T SNP in intron 1 and A+49G SNP in exon 1 of the CTLA-4 gene with T1D were investigated in 396 Egyptian patients ≤14 years old and 396 control subjects >24 years old, with the same ratio of males to females in both groups. The diagnosis of T1D was made on the basis of ketoacidosis or ketosis with severe symptoms of acute onset at presentation and continuous dependence on insulin. Controls were negative for anti-GAD antibodies and were greater than 24 years of age. Genotyping was performed using single strand conformation polymorphism (SSCP), temperature gradient gel electrophoresis (TGGE), and polymerase chain reaction-restriction fragment length polymorphism (PCR-RFLP).

**RESULTS:**

The results demonstrated an association of the C-819T and A+49G SNPs in the CTLA-4 gene with T1D patients (*P*=0.0047) and (*P*=0.000575), respectively. Moreover, this association was stratified by gender and age to female patients with age at onset 0-5 years old (*P*=0.0186) and (*P*=0.00115) more than male patient with the age at onset 0-5 years old (*P*= 0.3120) and (*P*=0.345161), respectively.

**CONCLUSION:**

The results support an association of the C-819T and A+49G SNPs in the CTLA-4 gene with Egyptian children, specifically, females of onset age 0-5 years old.

## Introduction

Type 1 diabetes (T1D) is a genetically complex disorder of glucose homeostasis that results from autoimmune destruction of the insulin-secreting cells of the pancreas. The development of T1D likely results from exposure to environmental factors which interact with a number of genes that contribute to the susceptibility of the disease.[1] Genetic susceptibility to T1D is conferred by more than 20 putative loci.[2] Approximately 40% of the susceptibility genes are located within the HLA locus on chromosome 6p21, known as IDDM1.[3] Another significant susceptibility locus (IDDM12) maps to the CTLA-4 gene region of chromosome 2q33.[4] IDDM12 has also been implicated in systemic lupus erythematosis, autoimmune thyroid diseases, coeliac disease, and rheumatoid arthritis, underscoring the importance of this locus in autoimmune processes.[5]

IDDM12 contains a cluster of T lymphocyte-regulating genes including CD28, CTLA-4, and ICOS. CD28 and CTLA4 are receptors that together with the antigen specific T-cell receptor-bind to the B7 family of receptors on the surface of antigen presenting cells. CD28 enhances whereas CTLA4 inhibits T-cell proliferation. Binding of CTLA4 to the B7 receptor limits the proliferation of T-cells and terminates the ongoing immune response.[6] CTLA4 knockout mice develop a severe lympho-proliferative disorder and die within a few days after birth, highlighting the importance of this gene in the negative regulation of the immune response.[7]

Increasingly, single nucleotide polymorphisms (SNPs) of the CTLA4 gene are correlated with autoimmune diseases. Most SNPs of the CTLA4 gene associated with autoimmune diseases are located within introns, with A+49G the only SNP found within an exon.[8] Overall, the data on the association of CTLA4 polymorphisms with T1D is convincing in some populations,[8–14] but not in others.[15–17] The identification of new SNPs for T1D is an important ongoing task.

In this study, we investigated whether the C-819T and A+49G SNPs of the CTLA4 gene are associated with T1D for the Egyptian population, a multi-ethnic group. We determined if expression of the C-819T and A+49G SNPs correlated with onset of T1D for Egyptian children and the prevalence of these polymorphisms with respect to gender.

## Materials and Methods

### Subjects

All individuals who participated in this study gave informed written consent. The study was approved by the Egyptian Bioethics Review Committee for Bioethics (BERD) - VACSERA. 396 patients with T1D from different governorates of Egypt were recruited. The diagnosis of T1D was made on the basis of ketoacidosis or ketosis with severe symptoms of acute onset at presentation and continuous dependence on insulin. All patients were non-obese and ≤14 years of age at enrolment. Patients were separated into two groups for statistical analysis: Less than six years of age (0-5 age group) and six to less than 15 years of age (6-14 age group). Healthy individuals (396) selected randomly from the Egyptian population to serve as controls were greater than 24 years of age, as recommended by the DiaMond protocol.[18] None of the controls had a family history of T1D. A standardized medical history questionnaire was completed by all patients and controls. The anti-GAD antibodies and C-peptide tests were performed to confirm the diagnosis of T1D for patients or to rule it out for controls. Individuals who were suspected to have Maturity-Onset Diabetes of the Young (MODY) or Wolfram syndrome were excluded from the study.

### SNP genotyping

Blood (1 ml) was collected in a tube containing EDTA, and the DNA was extracted by a salting out method.[19] The DNA was dissolved in 50 μl of Tris-EDTA (20 mM Tris, 2 mM EDTA, pH 7.5) buffer and stored at -20°C until use.

### Single strand conformation polymorphism (SSCP)

A 180 bp fragment in which the polymorphic site -819 in introns 1 of the CTLA gene was located in a central position was amplified by PCR using the following primers: Forward, CTLA4F_(-819)_-5’-GGAGAGGGGCCTGGTTAGTTACA-3’; Reverse, CTLA4R_(-819)_- 5’ AGAGAGGCAGCGGTG GTGTCA-3’. The sequence of C and T alleles published in NCBI with numbers EU103999 and EU104000, respectively. The reaction mixture contained 1 μl of target DNA (50-100 ng/ul DNA), 1.5 mM MgCl_2_, thermophilic DNA polymerase buffer (50 mM KCl, 10 mM Tris-HCl, pH 9.0, 0.1% Triton X-100), 0.2 mM of the four deoxynucleotide triphosphates, 0.2 unit of Taq polymerase and 10 ρmol of each primer in a total volume of 25 μl. PCR amplification was performed using a model PTC100 thermal cycler (MandJ research, Watertown, MA, USA) as follows: Denaturation at 94°C for 5 min, followed by 35 cycles of 30 seconds at 94°C, 40 sec at 62°C, and 40 sec at 72°C, and a final extension step for 10 min at 72°C.

While the 216 bp fragment for SSCP runs in which the polymorphic site +49A/G of the CTLA gene was located in a central position was amplified by PCR using the following primers: Forward, CTLA4F_(+49PCR-SSCP)_-5’- GAA CAC CGC TAG CCC ATA AA -3’; Reverse Primer, CTLA4R_(+49PCR-SSCP)_-5’- AAT CAC TGC CCT TGA CTG CT-3’. The sequence of A and G alleles published in NCBI with numbers DQ534199 and DQ534200, respectively. PCR amplification was performed using the same conditions above as follows: Denaturation at 94°C for 5 min, followed by 35 cycles of 30 sec at 94°C, 30 sec at 54°C, and 30 sec at 72°C, and a final extension step for 10 min at 72°C.

To identify single strand conformation polymorphism (SSCP), 5 μl of PCR product was mixed with 5 μl of loading dye (95% deionized formamide and 0.025% methylene blue), incubated for five minutes at 95°C, immersed in ice for 10 min, and loaded into an 8% polyacrylamide gel. Gel electrophoresis was performed at 240 volts over four hours at room temperature. Silver staining revealed variant mobility of conformational fragments bands, corresponding to nucleotide substitution.[20]

### Temperature gradient gel electrophoresis (TGGE)

TGGE was performed on PCR products carrying the SNP-819C/T and A+49G mutations, respectively and further amplified by PCR using the previously described conditions and non-GC-clamped PCR primers.[21] The PCR product (20 μl) was mixed with 20 μl of 4 M urea and 0.05% methylene blue, heated at 95°C for 5 min, and 50°C for 20 min. Samples were loaded into 1 mm polyacrylamide gels (5%) covalently bound to polyethylene gel support films (BioRad, Hercules, CA, USA) equilibrated with 1.25× TBE buffer. After electrophoresis, bands of heteroduplex and homoduplex DNA were electrophorezed overnight at 40 volts, ramping the temperature from 25 to 75°C (2 ± 0.5°C/h).[22] DNA was stained with ethidium bromide (0.01 g/L for 45-60 sec) and visualized using a UV transilluminator.

### Polymerase chain reaction- restriction fragement length polymorphism (PCR-RFLP)

For PCR-RFLP, PCR products (10 μl) were incubated overnight at 37°C with 2 units of the restriction enzyme CviKl-1 (New England Biolabs, UK). The digested PCR products were separated on a 2% agarose gel and visualized by EtBr staining. PCR products digested with the CviKl-1 restriction enzyme yielded two bands 69 bp and 111 bp in length for the CC allele, and three bands 180 bp, 111 bp, and 69 bp in length for the CT allele, whereas the TT allele was not cut by the restriction enzyme, and only one band 180 bp was evident.

A 152 bp (PCR-RFLP) fragment containing the +49 A/G polymorphism in exon 1 of *CTLA4* was amplified using a forward primer (CTLA4F)_+49PCR-RFLP_: *5’-AAGGCTCAGCTGAACCTGGT-3’* and a reverse primer (*CTLA4*R)_*+49PCR-RFLP*_: *5’-CTGCTGAAACAAATGAAACCC-3’.* The forward primer was designed with a single base mismatch for the last nucleotide, which corresponds to the +47 position, to introduce a base change in the sequence of the PCR product. The substitution creates a *Ecor911 (Fermantas, Lu)* restriction site in the A allele. Amplification was performed using the following conditions. Samples were subjected to 5 min at 94°C then 35 cycles of 30 sec at 94°C for denaturing, 30 sec at 55°C for annealing and 30 sec at 72°C for extension.

### Statistical analysis

Differences in genotype and allele distribution in cases and controls were tested for significance by the Chi-squared test with Yates’ continuity correction. Statistical significance was defined as *P*<0.05, achieved when the calculated χ^2^>3.841 for one degree of freedom, and χ^2^>5.991 for two degrees of freedom. Hardy-Weinberg equilibrium was tested by comparing the expected and observed genotypes in 2 × 3 χ^2^ tables. Odds ratio (OR) was calculated for allele distributions using the statistical program EpiCalc2000, http://www.brixtonhealth.com/epicalc.htm. No adjustments to the level of significance were made for multiple analyses.

## Results

The 396 T1D patients included 198 males and 198 females, a ratio of 1:1 [Table 1]. The 396 healthy individuals selected randomly from the Egyptian population to serve as controls included 198 males and 198 females, also a ratio of 1:1 to facilitate the statistical calculations to avoid any bias toward specific gender in the study. All controls were negative for anti-GAD antibodies and were 25 years of age or older, as recommended by the DiaMond protocol.[18] This age restriction is placed on the controls to exclude subjects from the high-risk period of 0-14 years of age, during which T1D is most likely to develop.[11] Furthermore, no control had a family history of T1D.

**Table 1:** Demographics of the study populations

Number of patients		Group I	Group II	All patients	Controls
		0-5 years old	6-14 years old	0-14 years old	>24 years old
	
		120	39	159	170
Age (yrs) ± Mean		3.8 ± 0.3	9.6 ± 0.5	6.7 ± 0.4	32.5 ± 3.7
Range		female 3.9±0.2	female 9.8 ± 0.3	0-14	25-39
		male 3.7±0.4	male 9.4 ± 0.7		
		0-5	6-14		
Weight (kg)		10.3 ± 1.2	29.7 ± 1.7	20.5± 1.45	73.4 ± 2.56
Range		2 ± 15.6	17.2 ± 44.8	2-44.8	65.5 ± 83.5
Sex, *N* (%)	Female	99 (50)	99 (50)	198 (50)	198 (50)
	Male	99 (50)	99 (50)	198 (50)	198 (50)
			Patient age groups		
Genotype		(0-5) *N*=198	(6-14) *N*=198	(0-14) *N*=396	(25-39) *N*=396
CC		88 (44.44%)	92 (46.46%)	180 (45.45%)	214 (54%)
CT		90 (45.45%)	88 (44.44%)	178 (44.95%)	164 (41.67%)
TT		20 (10.11%)	18 (9.10%)	38 (9.60%)	18 (4.33%)
Allele C		266 (0.672)	272 (0.687)	536 (0.677)	592 (0.747)
Allele T		130 (0.328)	124 (0.313)	256 (0.323)	200 (0.253)
Statistical Analysis^a^ Parameter			Patient age group 0-5	Patient age groups 6-14	Patient age groups 0-14
Genotype		χ^2,a^	9.2631	6.2563	10.65
		*P* value	0.0096	0.0437	0.0047
Allele		χ^2,b^	7.5524	4.8889	9.6573
		*P* value	0.005993	0.02703	0.001886
		Odds Ratio	0.690846	0.740277	0.708243

The difference in the disease onset age for female T1D patients (3.9 ± 0.2) versus male T1D patients (3.7 ± 0.4) in the 0-5 age group was not statistically significant [Table 1]. Similarly, the difference in the disease onset age for female T1D patients (9.8 ± 0.3) versus male T1D patients (9.4 ± 0.7) in the 6-14 age group was not statistically significant. The disease onset ages for the non-stratified age groups (female + male) were 3.8 ± 0.3 for the 0-5 age group, 9.4 ± 0.7 in the 6-14 age group and 6.7 ± 0.4 for the 0-14 age group, respectively.

The genotype distributions of the patient and control groups were tested for deviations from Hardy-Weinberg equilibrium (HWE). Neither patient nor control group deviated from HWE, as statistical significance for 2 × 3 table's calculation.

Statistically significant difference existed in the distribution of the C-819T and A+49G SNPs for the CTLA4 genotype between the 0-5 age group and healthy controls (*P*=0.0096, χ^2^= 9.2631) and (*P*=0.001185, χ^2^= 13.4755) more between the (6-14) age group and healthy controls (*P*=0.0437, χ^2^= 6.2563) and (*P*=0.023487, χ^2^= 7.5026). Moreover, the statistically significant difference between the (0-14) age group and healthy controls presents (*P*=0.0047, χ^2^= 10.65) and (*P*=0.000575, χ^2^= 14.9226) [Tables 2 and 4], respectively.

**Table 2:** Distribution of the CTLA-4 C-819 T SNP polymorphism gene in the Egyptian population

χ^2^ and *P* values were calculated by Chi-square test on stratified 2 × 2 table
^a^Compared to controls
^b^Continuity corrected

**Table 3:** Gender-related distribution of the CTLA-4 C-819 T SNP polymorphism in the Egyptian population

Genotype	Female patient age groups			Female Controls	Male patient age group			Male Controls
				
	0-5	6-14	0-14	25-39	0-5	6-14	0-14	25-39
	(*N*=99)	(*N*=99)	(*N*=198)	(*N*=198)	(*N*=99)	(*N*=99)	(*N*=198)	(*n*=198)
CC	38 (38.38%)	41 (41.41%)	79 (39.9%)	104 (52.52%)	50 (50.50%)	51 (51.51%)	101 (51.01%)	110 (55.56%)
CT	49 (49.49%)	46 (46.46%)	95 (48%)	84 (42.42)	41 (41.41%)	42 (42.42%)	83 (41.92%)	80 (40.40%)
TT	12 (12.13%)	12 (12.13%)	24 (12.1%)	10 (5.06%)	8 (8.09%)	6 (6.07%)	14 (6.57%)	8 (4.04%)
Allele C	125 (63.13%)	128 (64.65%)	253 (63.9%)	292 (73.74%)	141 (71.21%)	144 (72.73%)	285 (71.97%)	300 (75.76%)
Allele T	73 (36.87%)	70 (35.35%)	143 (36.1%)	104 (26.26%)	57 (28.79%)	54 (27.27%)	111 (28.03%)	96 (24.24%)
			(0-5) Females	(6-14) Females	(0-14) Females	(0-5) Males	(6-14) Males	(0-14) Males
Statistical Analysis^a^ parameter								
			(*N*=99)	(*N*=99)	(*N*=198)	(*N*=99)	(*N*=99)	(*N*=198)
Genotype	χ^2^		7.952	6.3697	9.8560	2.3290	1.8358	2.0755
	*P* value		0.0186	0.041385	0.0071	0.3120	0.6587	0.3542
Allele	χ^2^		7.0982	4.190	8.9487	1.4262	0.6422	1.4716
	*P* value		0.007716	0.021734	0.00277	0.2323	0.4229	0.2250
	Odds ratio		0.610184	0.651108	0.63211	0.788979	0.849592	0.822912

χ^2^ and *P* values were calculated by Chi-square tests

a Compared to controls

b Continuity corrected

**Table 4:** Distribution of the CTLA-4 A+49G SNP polymorphism gene in the Egyptian population

	Patient age groups			Controls
		
Genotype	0-5 age group, *N*=198	6-14 age group, *N*=198	0-14 age group, *N*=396	25-39 age group, *N*=396
AA	79 (39.90%)	87 (43.94%)	166 (41.92%)	215 (54.29%)
AG	90 (45.45%)	85 (42.93%)	175 (44.20%)	150 (37.88%)
GG	29 (14.65%)	26 (13.13%)	55 (13.88%)	31 (7.83%)
Allele A	248 (0.626)	259 (0.654)	507 (0.640)	580 (0.732)
Allele G	148 (0.347)	137 (0.346)	285 (0.360)	212 (0.268)
Statistical Analysis^a^ Parameter		Patient age group (0-5)	Patient age groups (6-14)	Patient age groups (0-14)
Genotype	χ^2,^^a^	13.4755	7.5026	14.9226
	*P* value	0.001185	0.023487	0.000575
Allele	χ^2,^^b^	14.0609	7.7980	15.6248
	*P* value	0.000177	0.00523	0.0000772
	Odds Ratio	0.612656	0.690713	0.65118

χ^2^ and P values were calculated by Chi-square test on stratified 2 × 2 table

a Compared to controls

b Continuity corrected

Similarly, statistically significant differences existed for the allele frequencies at position -819 in intron 1 and +49 in exon 1 between the 0-5 age group and healthy controls (*P*=0.005993, χ^2^=7.5524) and (*P*=0.000177, χ^2^=14.0609) is more than the 6-14 age group and healthy controls (*P*=0.02703, χ^2^=4.8889) and (*P*=0.00523, χ^2^=7.7980). Moreover, the statistically significant difference between the 0-14 age group and healthy controls (*P*=0.001886, χ^2^=9.6573) and (*P*=0.0000772, χ^2^=15.6248) [Tables 2 and 4], respectively.

Stratification of the data according to gender revealed statistically significant differences in genotype distribution of the C-819T and A+49G for females between the 0-14 age group and controls (*P*=0.0071, χ^2^=9.856) and (*P*=0.000373, χ^2^=15.7863) and females between the 0-5 age group and controls (*P*=0.0186, χ^2^=7.952) and (*P*=0.00115, χ^2^=13.5975), whereas females between the 6-14 age group and controls (*P*=0.041385, χ^2^=6.3697) and (*P*=0.02201, χ^2^=7.6325) stratified by gender differed less significantly from the respective control. While, the males between 0-14 age group and controls (*P*=0.3542, χ^2^=2.0755) and (*P*=0.30141, χ^2^=2.3986), males between the 0-5 age group and controls (*P*=0.312, χ^2^=2.329) and (*P*=0.345161, χ^2^=2.1275) and the males between the 6-14 age group and controls (*P*=0.6587, χ^2^=1.8358) and (*P*=0.520627, χ^2^=1.3054) revealed statistically non significant; Tables 3 and 5, respectively. Similarly, statistically significant differences existed for the allele frequencies at position -819 in intron 1and +49 in exon 1 for females between the 0-14 age group and controls (*P*=0.002777, χ^2^=8.9487) and (*P*=0.0000433, χ^2^=16.7202), females between the 0-5 age group and controls (*P*=0.007716, χ^2^=7.0982) and (*P*=0.000142, χ^2^=14.4788) and females between the 6-14 age group and controls (*P*=021734, χ^2^=4.190) and (*P*=0.004499, χ^2^=8.0707), whereas the difference for males between 0-14 age group and controls (*P*=0.2250, χ^2^=1.4716) and (*P*=0.119318, χ^2^=2.4262), males between the 0-5 age group and controls (*P*=0.2323, χ^2^=1.4262) and (*P*=0.141224, χ^2^=2.1646) and males between the (6-14) age group and controls (*P*=0.4229, χ^2^=0.6422) and (*P*=0.274318, χ^2^=1.1950), respectively were not statistically significant; Tables 3 and 5, respectively.

**Table 5:** Gender-related Distribution of the CTLA-4 A+49G SNP Polymorphism in the Egyptian population

	Female Patient age groups			Female Controls	Male Patient age group			Male Controls
				
	0-5	6-14	0-14	25-39	0-5	6-14	0-14	25-39
Geno type	(*N*=99)	(*N*=99)	(*N*=198)	(*N*=198)	(*N*=99)	(*N*=99)	(*N*=198)	(*n*=198)
AA	34 (34.34%)	39 (41.41%)	73 (36.87%)	109(55.05%)	45 (45.45%)	48 (48.48%)	93 (46.97%)	106 (53.54%)
AG	47 (47.47%)	45 (45.45%)	92 (46.46%)	73 (36.87%)	43 (43.43%)	40 (40.40%)	83 (41.92%)	77 (38.89%)
GG	12 (18.19%)	15 (15.16%)	34 (16.67%)	16 (8.08%)	11 (11.12%)	11 (11.22%)	22 (11.11%)	15 (7.57%)
Allele A	115 (0.58)	123 (0.621)	238 (0.598)	291 (0.735)	133 (0.672)	136 (0.687)	269 (0.679)	289 (0.730)
Allele G	83 (0.42)	75 (0.379)	160 (0.402)	105 (0.265)	65 (0.328)	62 (0.313)	127 (0.321)	107 (0.270)
			0-5 Females	6-14 Females	0-14 Females	0-5 Males	6-14 Males	0-14 Males
Statistical Analysis^a^ parameter								
			(*N*=99)	(*N*=99)	(*N*=198)	(*N*=99)	(*N*=99)	(*N*=198)
Genotype	χ^2^		13.5975	7.6325	15.7863	2.1275	1.3054	2.3986
	*P* value		0.00115	0.02201	0.000373	0.345161	0.520627	0.30141
Allele	χ^2^		14.4788	8.0707	16.7202	2.1646	1.1950	2.4262
	*P* value		0.000142	0.004499	0.0000433	0.141224	0.274318	0.119318
	Odds ratio		0.501305	0.592286	0.538884	0.756113	0.809852	0.78556

χ^2^ and *P* values were calculated by Chi-square tests

a Compared to controls

b Continuity corrected

## Discussion

This study demonstrated an association of the C-819T and A+49G SNPs in intron 1 and exon 1 of the CTLA-4 gene with T1D disease, respectively. This association was stratified by onset age to the 0-5 age group. A similar association stratified by onset age to the 0-5 age group has been reported for other ethnic groups.[23,24] The association of the C-819T and A+49G SNPs of the CTLA-4 gene with T1D was also stratified to females in our study, supporting a role for the C-819T and A+49G SNPs in the pathogenesis of T1D by a gender-specific mechanism.

The specific mechanism whereby the C-819T and A+49G SNPs of the CTLA-4 gene promote autoimmunity in females and lead to the development of T1D is unknown. Steroid hormones are considered the most likely factors that trigger the onset of female gender-stratified, genetically-based autoimmune diseases. This idea is reinforced by the increased prevalence of autoimmune diseases in women, the sexual dimorphism of the immune response, and the *in vitro* modulatory effects of sex steroids on immune functions. These modifiers could directly or indirectly target steroid receptors that act as transcription factors for the susceptibility genes associated with T1D, although no such regulatory role for sex hormones has been identified. However, this possibility is not without precedence, as sex hormones may act as critical modulatory factors that can induce disease expression.[25] The observation that the T_-819_ and G_+49_ allele's frequency was greater for T1D patients in the 0-5 onset age group suggests that this group experienced a stronger immune response than the 6-14 onset age group in the Egyptian children.

This study was not properly powered to prove statistically significant associations between the SNPs C-819T and A+49G of the CTLA-4 gene and T1D patients among the different age groups and sex. Rather, the study was designed to evaluate an overall association present between the C-819T and A+49G SNPs of the CTLA-4 gene and patients with T1D, regardless of age group and sex. However, the findings suggest that specifically targeted follow-up studies could yield confirmatory outcomes in different subsets of interest. Regarding the issue of study power, at least 544 patients in total (272 per group) would need to be evaluated to confirm the results of the current study at 80% power.

In summary, a strong association of the C-819T and A+49G SNPs in the CTLA-4 gene was found in the 0-5 onset age group for Egyptian female patients with T1D but not for male patients in the same age group. Currently, a gender preference is not evident in the Egyptian society, but future studies will clarify the demographics as well as the etiology of T1D for the C-819T and A+49G SNPs of the CTLA-4 gene in this population.
